# Realization of ferromagnetic graphene oxide with high magnetization by doping graphene oxide with nitrogen

**DOI:** 10.1038/srep02566

**Published:** 2013-09-02

**Authors:** Yuan Liu, Nujiang Tang, Xiangang Wan, Qian Feng, Ming Li, Qinghua Xu, Fuchi Liu, Youwei Du

**Affiliations:** 1 Physics Department & Nanjing National Laboratory of Microstructures, Nanjing University, Nanjing 210093, P. R. China

## Abstract

The long spin diffusion length makes graphene very attractive for novel spintronic devices, and thus has triggered a quest for integrating the charge and spin degrees of freedom. However, ideal graphene is intrinsic non-magnetic, due to a delocalized π bonding network. Therefore, synthesis of ferromagnetic graphene or its derivatives with high magnetization is urgent due to both fundamental and technological importance. Here we report that N-doping can be an effective route to obtain a very high magnetization of ca. 1.66 emu/g, and can make graphene oxide (GO) to be ferromagnetism with a Curie-temperature of 100.2 K. Clearly, our findings can offer the easy realization of ferromagnetic GO with high magnetization, therefore, push the way for potential applications in spintronic devices.

Chemical doping is a fascinating way to intrinsically modify and improve graphene's physical and chemical properties. Notably, among the potential dopants, N atom is considered as an excellent candidate because it has comparable atomic size and contains five valence bonds with carbon atoms^1^. Nowadays, N-doped graphene (NG) has attracted much attention because of its excellent performance in electrocatalytic activity, electrical, fuel cells, and optical properties, *etc*^2^,^3^,^4^. Notably, magnetism of graphene and its derivatives is of particular interest since the light weight magnets could open up new ways to design adaptable and flexible information storage systems^5^. Especially, the greatly potential application of graphene-based magnets in spintronics is promising, since graphene has extraordinary carrier mobility and may provide an easy way to integrate spin and molecular electronics^6^. The long spin diffusion lengths and coherent times arising from the weak spin-orbit and hyperfine interactions in graphene can provide ideal conditions for coherent spin manipulation which can act as the next-generation spintronic devices^7^. However, graphene is usually intrinsic non-magnetic and lacks of localized magnetic moments due to a delocalized π bonding network, which limits its applications in spintronic devices^8^.

Generally, point defects such as vacancies, zigzag edges, chemical doping of foreign atoms can induce localized magnetic moments in graphene, which is the preliminary of the existence of magnetic ordering^9^,^10^,^11^,^12^,^13^,^14^,^15^,^16^,^17^,^18^. It is demonstrated that high-density vacancies can make graphene fragile and lose its structural stability, by contrast, chemical doping can keep its structural stability and can introduce more point defects in graphene^19^,^20^. For example, fluorination of graphene can obtain a high magnetization up to 0.2 emu/g, one order higher than the maximum value of 0.02 emu/g induced by vacancies^20^. Up to now, the reported magnetic moment of graphene is still low, and the distance between magnetic moments is long. As a result, these graphene materials always exhibit typically Curie-like paramagnetism^20^,^21^,^22^. Therefore, to combine charge and spin manipulation for the novel spintronic devices, it is urgent to develop effective methods for sythesizing ferromagnetic graphene with high magnetization. Note that, graphene oxide (GO) is usually considered as an insulator or a semiconductor. However, after chemical or thermal reduction, it can be zero-band-gap semi-metal graphene nanosheets^23^. Actually, after efficient reduction, the achieved mobility value of 5000 cm^2^ V^−1^ s^−1^ is close to that of mechanically cleaved graphene (ca. 10000 cm^2^ V^−1^ s^−1^)^24^. It has been demonstrated that annealing in ammonia can effectively reduce GO^3^, indicating its potential in spintronics.

Theoretical study confirmed that N-doping is an effective method to introduce magnetic moments into graphene^25^,^26^,^27^,^28^,^29^. In this study, we demonstrate that doping GO with N can obtain a very high magnetization (1.66 emu/g) and a significant increase of magnetization of GO (ca. 1509.1%). Most importantly, it makes the magnetism of GO from purely spin-half paramagnetism to ferromagnetism with Curie temperature (*T*_C_) of ca. 100.2 K.

## Results

### Microstructures of GO and NGO

**Figure 1:**
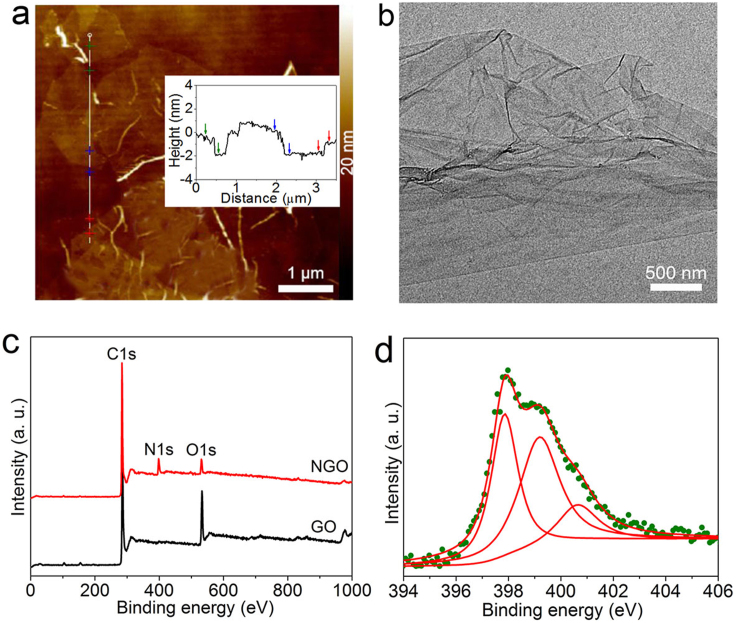
Microstructure of GO and NGO. (a) Tapping mode AFM height image of GO. Inset: The corresponding height profiles of the white lines with colorful crosses. The colorful arrows correnspond to the colorful crosses. (b) TEM image of GO. (c) XPS spectra of GO and NGO over a wide range of binding energies (0–1000 eV). (d) Fine-scanned N 1s XPS spectrum of NGO. The olive dots and red lines are measured dots and fitting curves.

We prepared N-doped graphene oxide (NGO) by directly annealing GO in ammonia at 500°C. For GO, two factors have important influence on N content: few-layer ratio and O content of GO. It has been reported that compared to multi-layer GO, few-layer GO is more sensible to the adatoms, and can be doped with higher N content^30^. It also has been confirmed that oxygen groups in GO favor the reactions with NH_3_ and C–N bond formation, and directly annealing GO in ammonia can obtain high-content NGO^31^. Thus, to get GO with high few-layer ratio^32^, we centrifugated GO solution at 6000 rpm for 10 minutes, and only collected GO dispersed in the brown supernatant for N-doping. To quantitatively analyze the layer number, we investigated GO by atomic force microscope (AFM). Shown in Fig. 1a is the typical AFM image. As indicated by the height images (inset of Fig. 1b), most of the heights of GO layers are ca. 1 nm. Based on the fact that the height may increase because of the trapped solvent and the folds between GO and the substrate, one can expect that most of the GO sheets are single- or bi-layer^6^. Shown in Fig. 1b is a typical transmission electron microscope (TEM) image of GO. One can find that the GO sheets are not well flat, and look like wrinkled or crumpled thin papers with the size of several micrometers. To detect the O content of GO, we performed the X-ray photoelectron spectrum (XPS) measurement. As shown in Fig. 1c, the XPS spectrum of GO indicates that O content of GO defined as 100 O/C at.% is 48.37 at.%, indicating that the O content is high. Namely, we have synthesized GO with high few-layer ratio and O content.

Moreover, to detect the contents of O and N and bonding environment of C and N species of NGO, we also performed the XPS measurement. As shown in Fig. 1c, the XPS spectrum of NGO reveals that the O content which also defined as 100 O/C at.% is 6.59 at.%, much lower than that of GO. It demonstrates that a considerable amount of oxygen-containing functional groups were removed during annealing, which may be due to the formation of C–N bonds and reduction effects of thermal annealing in ammonia^3^,^31^. Moreover, it is found that compared to GO, NGO shows a clear N signal at ca. 400 eV. Shown in Fig. 1d is the high-resolution XPS spectrum in the N 1s binding energy range of NGO, which was deconvoluted into three subpeaks. The three subpeaks located at 398.3, 400.0, and 401.4 eV are attributed to pyridinic-like (N-6), pyrrolic-like (N-5), and graphitic-like (N-Q) atoms, respectively^3^,^33^. Based on the XPS results, the content of the N defined as 100 N/C at.% of NGO can be calculated to be 8.80, and the contents of N-6, N-5, and N-Q types are 3.45, 4.00, and 1.35 at.%, respectively. In other words, we have synthesized NGO with high N content by annealing GO with high few-layer ratio and O content.

### Analysis of the spin-half paramagnetism of GO

**Figure 2:**
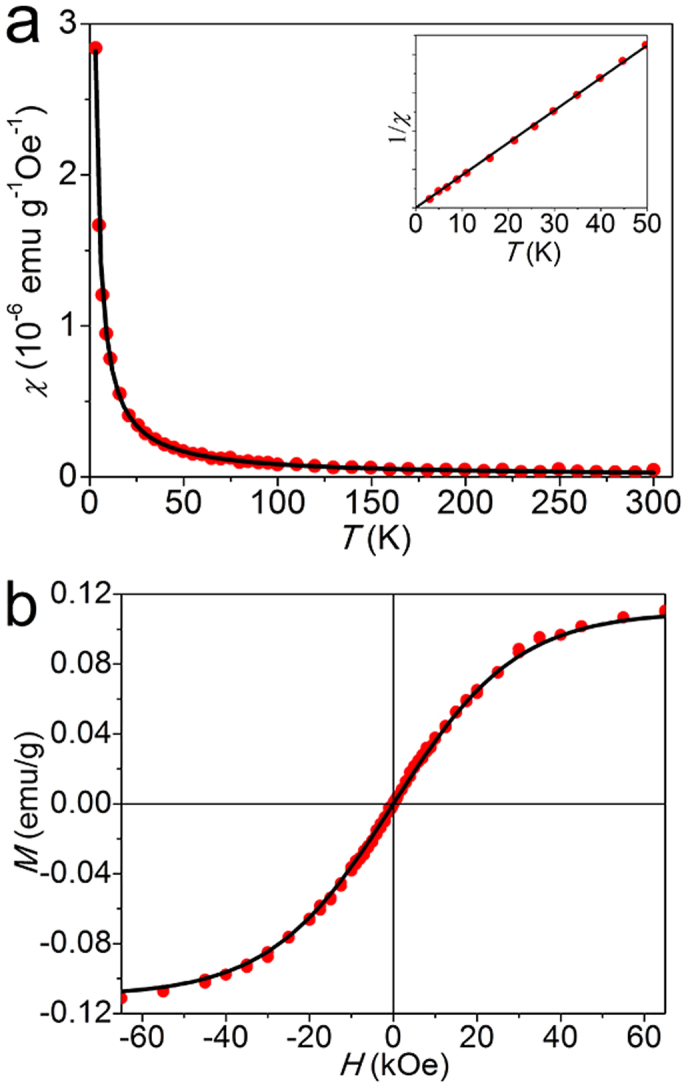
Magnetic properties of GO measured by a superconducting quantum interference device (SQUID) magnetometer. (a) Typical *χ* − *T* curve measured from 2 to 300 K under the applied field *H* = 3 kOe. Inset is the corresponding 1/*χ* − *T* curve. Red symbols are the measurements and black solid line is fitted by the Curie law. (b) *M* − *H* curve measured at 2 K. Red symbols are the measurements and black solid curve is fit to the Brillouin function with g = 2 and *J* = 1/2.

We first carefully measured magnetic properties GO. Shown in Fig. 2a is the dependence of susceptibility χ = *M*/*H* on temperature *T* and it fits well with the Curie law χ = *C*/*T*. Inset is the corresponding 1/*χ* − *T* curve, which demonstrates a linear, purely Curie-like paramagnetic behavior, a solid evidence of the existence of localized magnetic moment. As shown in Fig. 2b, no significant ferromagnetic signal is observed even at 2 K. The *M* − *H* curve is well fitted using the Brillouin function 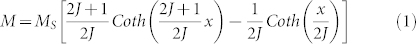
Where *x* = *gJμ_B_H*/(*k_B_T*), *M*_S_ = *NgJμ_B_*, *k_B_* the Boltzmann constant, *N* the number of present magnetic moments, *J* the angular momentum number, and g is the Landau factor which is assumed to be 2. As shown by the fitting curve, the Brillouin function provides good fits for *J* = *S* = 1/2. The intrinsic spin-half paramagnetism agrees well with the existing theories for the contributions of point defects such as vacancies, adatoms, and edges^20^,^34^,^35^. Also by fitting the curve, *M_s_* can be obtained which is 0.11 emu/g, similar to the value reported^21^.

### Magnetic properties of NGO

**Figure 3:**
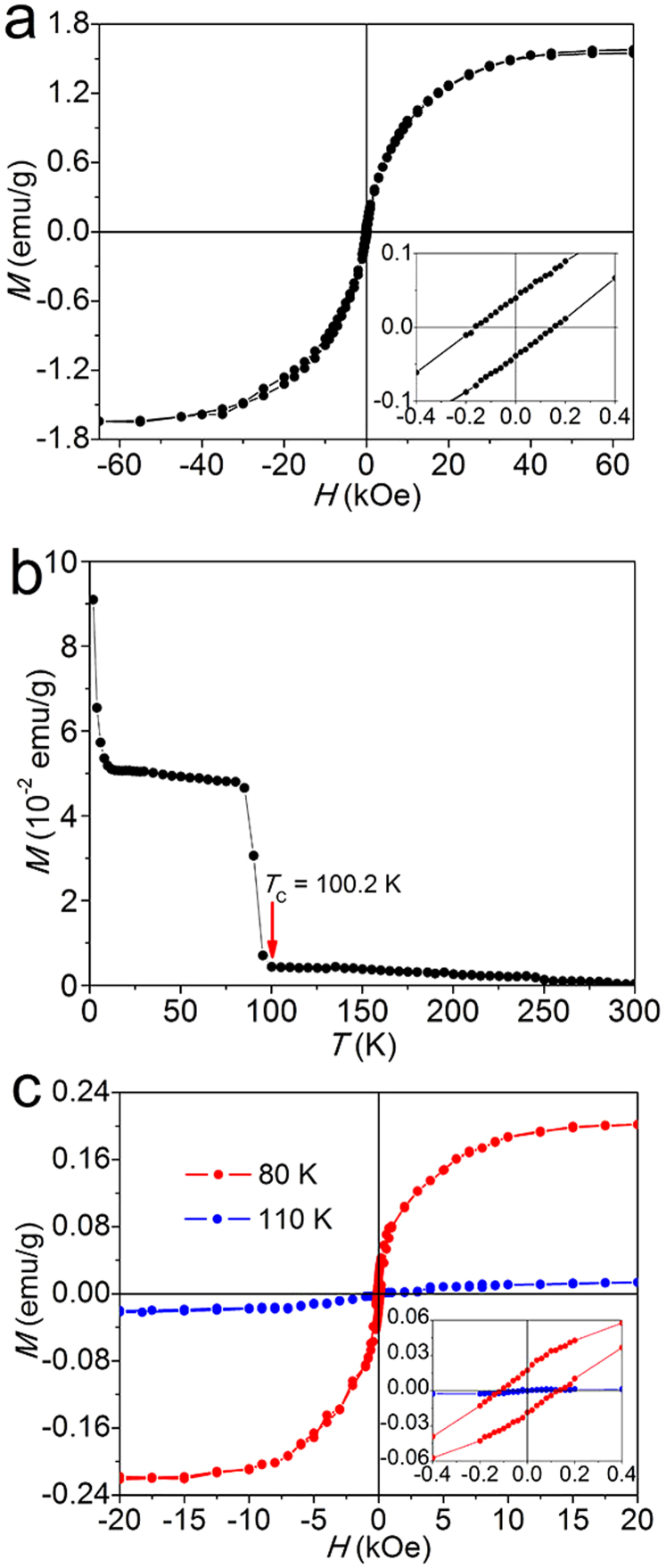
Magnetic properties of NGO measured by SQUID. (a) Typical *M* − *H* curve measured at 2 K. Inset is a part of the magnetization curve. (b) *M* − *T* curve of NGO measured from 2 to 300 K under the applied field *H* = 500 Oe. (c) *M* − *H* curves of NGO measured at 80 and 110 K. Inset is a part of the magnetization curves.

Subsequently, we carried out the magnetic measurements for NGO. Note that no positive magnetic signal, and only purely diamagnetism can be observed in both GO and NGO at 300 K (Supplementary Fig. S1). Figure 3a shows the typical mass magnetization (*M* − *H*) curve of NGO measured at 2 K. One can find that the coercive field (*H*_c_) and remnant magnetization (*M*_r_) are 160 Oe and 0.039 emu/g (inset of Fig. 3a), a solid evidence for ferromagnetism. Subsequently, we performed the *M* − *T* measurement of NGO and found a clear *T_C_* at ca. 100.2 K (Fig. 3b), implying that the *T_c_* is above liquid N_2_ temperature of 77 K. It also can be confirmed by the two *M* − *H* curves measured at 80 and 110 K (Fig. 3c).

**Figure 4:**
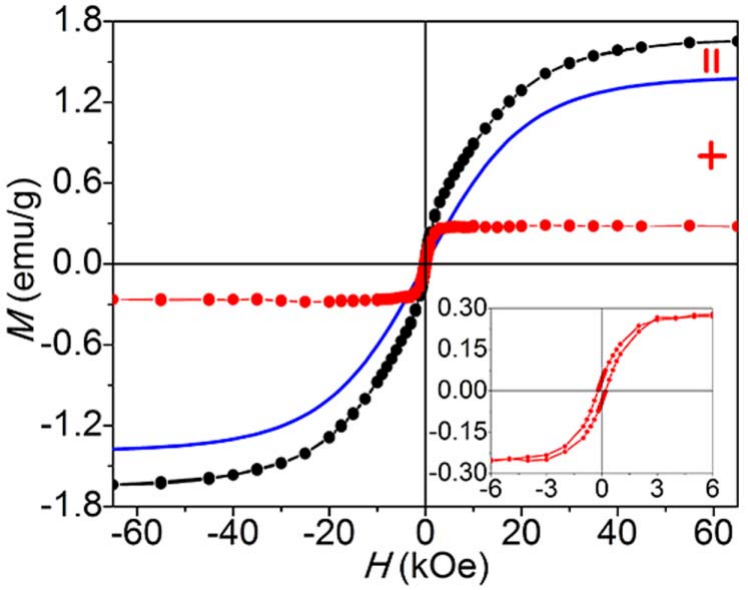
Analysis of the 2 K *M* − *H* curve of NGO. Black curve is the measured curve, and blue line is the fitting curve for paramagnetism by the Brillouin function with g = 2 and *J* = 1.11. Red curve is ferromagnetic mass magnetization by subtracting the paramagnetism from the measured curve. Inset is a part of the ferromagnetic mass magnetization.

It is found that the *M* − *T* curve of NGO shows typically paramagnetic behavior at low temperature below 8 K (Fig. 3b). Namely, the magnetism at 2 K is composed of two parts: paramagnetism and ferromagnetism, which can be expressed as *M*_total_ = *M*_para_ + *M*_ferro_. Considering the fact that ferromagnetic mass magnetization can saturate at a high applied field, one have to set paramagnetic *M*_0_ of the sample as 1.39 emu/g and *J* as 1.11 to fit the curve by Brillouin function (Fig. 4). As reported, *J* can be 0.5, 1, or 2.5, *etc*., which generally should be the integral multiple of 0.5, and corresponds to magnetically coupled unpaired electrons^20^,^21^,^22^. However, the magnetic structure of our sample may be uneven, and different *J* may exist in different sheets in NGO. For example, some of sheets show *J* = 0.5, and some show *J* = 1, or 1.5. Therefore, as an average, the total *J* may become 1.11. Moreover, theoretically, the N atom can induce a net magnetic moments of 1.8, 0.95, or 0.67 *μ*_B_ for specific structures^25^,^29^. It also may be the reason that *J* = 1.11 in NGO. By subtracting the paramagnetic signal from the observed data, one can obtain the remaining ferromagnetic moment. The magnetization is approximately saturated at 6 kOe (inset of Fig. 4), and the saturation magnetization of ferromagnetic signal is ca. 0.27 emu/g at 2 K. We should note that the issue of the minute magnetic contaminant in the sample is very important because trace ferromagnetic impurites also can contribute to the ferromagnetic signal observed^36^,^37^. Therefore, we have very carefully analyzed the magnetic impurities of NGO by inductively coupled plasma (ICP) analysis (Supplementary Table S1). Clearly, the results showed that the magnetic impurity elements (such as Fe, Co, Ni or Mn) of NGO are below 10 ppm, demonstrating that NGO is highly pure, and the contribution of magnetic impurities is neglectable. Actually, the ferromagnetic signal of ca. 0.27 emu/g is equivalent to 1277 ppm Fe, ca. 284 times higher than the ICP result. Clearly, the contribution of magnetic impurities is neglectable. From *M*_0_ added with saturated ferromagnetic magnetization, one can calculate the *M*_s_ of NGO is ca. 1.66 emu/g. To best of our knowledge, it is the highest intrinsic magnetization reported for all graphene materials. Interestingly, N-doping increases the magnetization of GO from 0.11 to 1.66 emu/g, a significant increase of ca. 1509.1%. It is clear that N-doping results in the increase of the magnetization of GO and the generation of ferromagnetism.

## Discussion

Actually, the contribution of different structural defects like vacancies and O for magnetization in GO and NGO may be different. Especially, the O content is relatively high, and the difference of O contribution in GO and NGO is highly possible^38^,^39^. Approximately, to make a simple comparison with the results of NGO, we obtained GO samples (GO-a300, GO-a500, GO-a700, and GO-a900, numeric numbers denote the annealing temperature) by annealing GO in Ar. Based on the XPS results, the O contents defined as 100 O/C at.% are in the range of 5.7–13.35 at.% in annealed GO samples (Supplementary Table S2). The magnetic results show that all of the annealed GO samples are spin-half paramagnetic with a low magnetization similar to GO (Supplementary Fig. S2), one order of magnitude lower than that of NGO. Thus, it is reasonable to assume that the contribution of O to the significant increase of the magnetization and the generation of ferromagnetism in NGO is negligible.

Theoretical calculation showed that localized magnetic moments can be induced by different types of N atoms^25^,^26^,^27^,^28^,^29^. As proposed by Zhang et al., a combination of vacancy defects and N atoms may provide a unique way for enhancing the magnetic moment^28^. Especially, N-5 atom plays the important role in the introduction of magnetic moments because a N-5 atom can introduce a net magnetic moment of 0.95 *μ*_B_ at either the edge or defect because of the contribution of the π bonds^25^,^27^. By contrast, N-Q atom was considered to support no magnetic moment, however, it may act as a stable attractor, forming a magnetic defect complex N-Q + C-ad, which can contribute magnetic moment of 0.98 *μ*_B_^26^. Considering that O content in our NGO is relatively high, the formation of N-Q + O-ad may be highly possible^29^. Thus, we cannot exclude the contribution from N-Q. We note that the specific contribution by each specific type of the three N types is complicated, and it is difficult to clarify the specific contribution in NGO. Moreover, the magnetic coupling between the localized magnetic moments is the preliminary of the existence of ferromagnetic ordering. Notably, after reduction, GO can transform from an insulator to a graphene-like semi-metal^40^. Thus, the magnetic coupling between the magnetic moments may appear via Ruderman–Kittel–Kasuya–Yosida (RKKY) interactions by delocalized π electrons, which is expected to decay as *D*^−*r*^, where *D* is the distance between the magnetic moments and *r* is the decay exponent^41^,^42^. It has been confirmed that graphene with low magnetization exhibits pure Curie-like paramagnetism due to the long distance between magnetic moments^20^,^21^. In our NGO, it is reasonable to assume that with the increasing of localized magnetic moments of GO from 3.89 to 42.48 *μ_B_* per 10000 C atoms because of N-doping, the distance between the magnetic moments will decrease. As to the decay exponent *r*, it has been proposed that with the enhancing of electron-electron interaction, *r* may decrease and the power-law decay becomes more long ranged^43^. Different from the case of fluorination which makes the Fermi level shift downward^19^,^44^, N-doping can make the Fermi level shifted upward due to the extra π electrons, and make graphene electron-rich^45^. Thus, N-doping can enhance the magnetic coupling between the magnetic moments because of the decrease of distance and decay exponent.

Despite that the RKKY interaction usually occurs in metallic material, the bound magnetic polarons (BMPs) may also generate ferromagnetism in semiconductors. In short, the ferromagnetic coupling is mediated by shallow donor electrons that form BMPs, which overlap to create a spin-split impurity band^46^. For example, oxygen vacancies can act as BMPs and trap free electrons, and the electrons trapped in these BMPs tend to get easily polarized under the influence of the magnetic field and, resulting in ferromagnetism^47^. Similarly, in NGO, the N atoms may act as BMPs thanks to the strong electron affinity^48^. We note that the exact ferromagnetic coupling mechanism is not clear at present, and need to be confirmed by more experimental and theoretical work.

Additionally, we obtained NGO-a700 and NGO-a900 by annealing NGO in Ar at 700 and 900°C, and found that annealing-dependence magnetic properties support this law. In short, annealing NGO resulted in the decreasing in N content (Supplementary Table S3), magnetiztion, and *T_c_* (Supplementary Figs. S3 & S4). The localized magnetic moments also can be induced by F or H atoms, however, H and F atoms have a strong tendency towards clustering or even lose at relatively low temperatures^44^,^49^. Our results indicates that NGO can has relatively high stability in both structure and magnetic properties, thus, N-doping provides a good candidate for H- and F-doping.

Anyway, we successfully demonstrated that N-doping can make GO to be ferromagnetic with a very high magnetization of 1.66 emu/g and an above liquid N_2_-temperature *T*_C_ of ca. 100.2 K. Thus, our method offers the easy fabrication of ferromagnetic GO with high magnetization, therefore, pushes the way for potential applications in magnetic graphene.

## Methods

### Sample preparation

GO was synthesized by chemical exfoliation of natural flake graphite powder (500 mesh). The solution of GO was first centrifugated at 6000 rpm for 10 min, and the brown supernatant was collected to get few-layer GO. To obtain highly pure GO sample, GO was washed with hydrochloric acid for 15 times and then with ethanol for 10 times. Thereafter, NGO was obtained by annealing GO at 500°C for 3 h in ammonia (~99%) under ambient pressure.

### Structural characterization and magnetic measurements

The morphologies of the samples were investigated by TEM (model JEM–2100, Japan) and AFM (Veeco dimension V, USA). For quantitative determination of the chemical composition and the bonding environment of the samples, XPS were measured on PHI5000 VersaProbe (ULVAC-PHI, Japan) using Al Ka radiation. The magnetic properties of the samples were measured using SQUID magnetometer with a sensitivity of less than 10^−8^ emu (Quantum Design MPMS–XL, USA), and all data have been corrected for the diamagnetic contribution by subtracting the corresponding linear diamagnetic background measured at RT. The magnetic impurity elements (such as Fe, Co, Ni or Mn) of all the samples are below 10 ppm measured by ICP spectrometry (Jarrell–Ash, USA).

## Author Contributions

N.J.T. proposed and supervised the project, Y.L. and N.J.T. designed the experiments, Y.L. carried out the experiments, Y.L., N.J.T. and X.G.W. analyzed the data and wrote the manuscript. Q.F., M.L., Q.H.X., F.C.L. and Y.W.D. had valuable discussions and edited the manuscript. All the authors commented on the manuscript.

## Supplementary Material

Supplementary Information Supplementary Information

## References

[b1] Lee S. U., Belosludov R. V., Mizuseki H. & Kawazoe Y. Designing nanogadgetry for nanoelectronic devices with nitrogen-doped capped carbon nanotubes. Small 5, 1769–1775 (2009). 19360721 10.1002/smll.200801938

[b2] Wang X. R. *et al.* N-doping of graphene through electrothermal reactions with ammonia. Science 324, 768–771 (2009). 19423822 10.1126/science.1170335

[b3] Li M. *et al.* Quenching of fluorescence of reduced graphene oxide by nitrogen-doping. Appl. Phys. Lett. 100, 233112 (2012).

[b4] Qu L. T., Liu Y., Baek J. B. & Dai L. M. Nitrogen-doped graphene as efficient metal-free electrocatalyst for oxygen reduction in fuel cells. ACS Nano 4, 1321–1326 (2010). 20155972 10.1021/nn901850u

[b5] Yang X. *et al.* Correlation between the vacancy defects and ferromagnetism in graphite. Carbon 47, 1399–1406 (2009).

[b6] Novoselov K. S. *et al.* Electric field effect in atomically thin carbon films. Science 306, 666–669 (2004). 15499015 10.1126/science.1102896

[b7] Tombros N., Jozsa C., Popinciuc M., Jonkman H. T. & van Wees B. J. Electronic spin transport and spin precession in single graphene layers at room temperature. Nature 448, 571–574 (2007). 17632544 10.1038/nature06037

[b8] Li L. Z. *et al.* Functionalized graphene for high-performance two-dimensional spintronics devices. ACS Nano 5, 2601–2610 (2011). 21395280 10.1021/nn102492g

[b9] Soriano D. *et al.* Magnetoresistance and magnetic ordering fingerprints in hydrogenated graphene. Phys. Rev. Lett. 107, 016602 (2011). 21797560 10.1103/PhysRevLett.107.016602

[b10] Uchoa B., Kotov V. N., Peres N. M. R. & Neto A. H. C. Localized magnetic states in graphene. Phys. Rev. Lett. 101, 026805 (2008). 18764214 10.1103/PhysRevLett.101.026805

[b11] Palacios J. J., Fernandez-Rossier J. & Brey L. Vacancy-induced magnetism in graphene and graphene ribbons. Phys. Rev. B 77, 195428 (2008).

[b12] Cervenka J., Katsnelson M. I. & Flipse C. F. J. Room-temperature ferromagnetism in graphite driven by two-dimensional networks of point defects. Nat. Phys. 5, 840–844 (2009).

[b13] Tada K. *et al.* Ferromagnetism in hydrogenated graphene nanopore arrays. Phys. Rev. Lett. 107, 217203 (2011). 22181918 10.1103/PhysRevLett.107.217203

[b14] Sun Q. *et al.* Ferromagnetism in semihydrogenated graphene sheet. Nano Lett. 9, 3867–3870 (2009). 19719081 10.1021/nl9020733

[b15] Yazyev O. V. Emergence of magnetism in graphene materials and nanostructures. Rep. Prog. Phys. 73, 056501 (2010).

[b16] Yazyev O. V. & Katsnelson M. I. Magnetic correlations at graphene edges: Basis for novel spintronics devices. Phys. Rev. Lett. 100, 047209 (2008). 18352331 10.1103/PhysRevLett.100.047209

[b17] Esquinazi P. *et al.* Induced magnetic ordering by proton irradiation in graphite. Phys. Rev. Lett. 91, 227201 (2003). 14683267 10.1103/PhysRevLett.91.227201

[b18] Ma Y. W. *et al.* Room temperature ferromagnetism in Teflon due to carbon dangling bonds. Nat. Commun. 3, 727 (2012). 22395618 10.1038/ncomms1689

[b19] Robinson J. T. *et al.* Properties of fluorinated graphene films. Nano Lett. 10, 3001–3005 (2010). 20698613 10.1021/nl101437p

[b20] Nair R. R. *et al.* Spin-half paramagnetism in graphene induced by point defects. Nat. Phys. 8, 199–202 (2012).

[b21] Sepioni M. *et al.* Limits on intrinsic magnetism in graphene. Phys. Rev. Lett. 105, 207205 (2010). 21231263 10.1103/PhysRevLett.105.207205

[b22] Ney A., Papakonstantinou P., Kumar A., Shang N. G. & Peng N. H. Irradiation enhanced paramagnetism on graphene nanoflakes. Appl. Phys. Lett. 99, 102504 (2011).

[b23] Wang S. *et al.* Band-like transport in surface-functionalized highly solution-processable graphene nanosheets. Adv. Mater. 20, 3440–3446 (2008).

[b24] Wang S. *et al.* High mobility, printable, and solution-processed graphene electronics. Nano Lett. 10, 92–98 (2009). 20025234 10.1021/nl9028736

[b25] Li Y. F., Zhou Z., Shen P. W. & Chen Z. F. Spin gapless semiconductor-metal-half-metal properties in nitrogen-doped zigzag graphene nanoribbons. ACS Nano 3, 1952–1958 (2009). 19555066 10.1021/nn9003428

[b26] Ma Y. C., Foster A. S., Krasheninnikov A. V. & Nieminen R. M. Nitrogen in graphite and carbon nanotubes: Magnetism and mobility. Phys. Rev. B 72, 205416 (2005).

[b27] Ma C. C., Shao X. H. & Cao D. P. Nitrogen-doped graphene nanosheets as anode materials for lithium ion batteries: A first-principles study. J. Mater. Chem. 22, 8911–8915 (2012).

[b28] Zhang Y. *et al.* First-principles study of defect-induced magnetism in carbon. Phys. Rev. Lett. 99, 107201 (2007). 17930406 10.1103/PhysRevLett.99.107201

[b29] Dai J. Y. & Yuan J. M. Adsorption of molecular oxygen on doped graphene: Atomic, electronic, and magnetic properties. Phys. Rev. B 81, 165414 (2010).

[b30] Novoselov K. S. *et al.* Detection of individual gas molecules adsorbed on graphene. Nat. Mater. 6, 652–655 (2007). 17660825 10.1038/nmat1967

[b31] Li X. L. *et al.* Simultaneous nitrogen doping and reduction of graphene oxide. J. Am. Chem. Soc. 131, 15939–15944 (2009). 19817436 10.1021/ja907098f

[b32] Wu Z. S. *et al.* Synthesis of high-quality graphene with a pre-determined number of layers. Carbon 47, 493–499 (2009).

[b33] Wei D. C. *et al.* Synthesis of N-doped graphene by chemical vapor deposition and its electrical properties. Nano Lett. 9, 1752–1758 (2009). 19326921 10.1021/nl803279t

[b34] Krasheninnikov A. V., Lehtinen P. O., Foster A. S., Pyykko P. & Nieminen R. M. Embedding transition-metal atoms in graphene: Structure, bonding, and magnetism. Phys. Rev. Lett. 102, 126807 (2009). 19392310 10.1103/PhysRevLett.102.126807

[b35] Yazyev O. V. Magnetism in disordered graphene and irradiated graphite. Phys. Rev. Lett. 101, 037203 (2008). 18764285 10.1103/PhysRevLett.101.037203

[b36] Sepioni M., Nair R. R., Tsai I. L., Geim A. K. & Grigorieva I. V. Revealing common artifacts due to ferromagnetic inclusions in highly oriented pyrolytic graphite. EPL 97, 47001 (2012).

[b37] Venkatesan M., Dunne P., Chen Y. H., Zhang H. Z. & Coey J. M. D. Structural and magnetic properties of iron in graphite. Carbon 56, 279–287 (2013).

[b38] Wang M., Huang W., Chan-Park M. B. & Li C. M. Magnetism in oxidized graphenes with hydroxyl groups. Nanotechnology 22, 105702 (2011). 21289406 10.1088/0957-4484/22/10/105702

[b39] Lehtinen P. O. *et al.* Magnetic properties and diffusion of adatoms on a graphene sheet. Phys. Rev. Lett. 91, 017202 (2003). 12906568 10.1103/PhysRevLett.91.017202

[b40] Eda G., Mattevi C., Yamaguchi H., Kim H. & Chhowalla M. Insulator to semimetal transition in graphene oxide. J. Phys. Chem. C 113, 15768–15771 (2009).

[b41] Power S. & Ferreira M. Indirect exchange and Ruderman–Kittel–Kasuya–Yosida (RKKY) interactions in magnetically-doped graphene. Crystals 3, 49–78 (2013).

[b42] Ruderman M. A. & Kittel C. Indirect exchange coupling of nuclear magnetic moments by conduction electrons. Phys. Rev. 96, 99–102 (1954).

[b43] Black-Schaffer A. M. Importance of electron-electron interactions in the RKKY coupling in graphene. Phys. Rev. B 82, 073409 (2010).

[b44] Nair R. R. *et al.* Fluorographene: A two-dimensional counterpart of Teflon. Small 6, 2877–2884 (2010). 21053339 10.1002/smll.201001555

[b45] Jalili S. & Vaziri R. Study of the electronic properties of li-intercalated nitrogen doped graphite. Mol. Phys. 109, 687–694 (2011).

[b46] Coey J. M. D., Venkatesan M. & Fitzgerald C. B. Donor impurity band exchange in dilute ferromagnetic oxides. Nat. Mater. 4, 173–179 (2005). 15654343 10.1038/nmat1310

[b47] Bhosle V. & Narayan J. Observation of room temperature ferromagnetism in Ga : ZnO: A transition metal free transparent ferromagnetic conductor. Appl. Phys. Lett. 93, 021912 (2008).

[b48] Gong K. P., Du F., Xia Z. H., Durstock M. & Dai L. M. Nitrogen-doped carbon nanotube arrays with high electrocatalytic activity for oxygen reduction. Science 323, 760–764 (2009). 19197058 10.1126/science.1168049

[b49] Openov L. A. & Podlivaev A. I. Thermal desorption of hydrogen from graphane. Tech. Phys. Lett. 36, 31–33 (2010).

