# Supplementary material for: Realization of ferromagnetic graphene oxide with high magnetization by doping graphene oxide with nitrogen
Source: Sci Rep. 2013 Sep 2;3:2566. doi: 10.1038/srep02566 (PMC3759045; doi:10.1038/srep02566)
Supplement: Supplementary Information [file srep02566-s1.pdf]

Supplementary Information for

"Realization of ferromagnetic graphene oxide with high magnetization by doping graphene oxide with nitrogen"

Yuan Liu[1], Nujiang Tang[1], Xiangang Wan[1], Qian Feng[1], Ming Li[1], Qinghua Xu[1], Fuchi Liu[1] & Youwei Du[1]

[1]Physics Department & Nanjing National Laboratory of Microstructures, Nanjing University, Nanjing 210093, PR China.

Supplementary Table S1 | The contents of the metal impurity elements (such as Fe, Co, Ni, or Mn) of all the samples measured by the ICP spectrometry. The unit is ppm, and 'ND' denotes 'not found'.

Samples (ppm)	Fe	Co	Ni	Mn
GO	9.4	ND	ND	3.7
NGO	4.5	ND	ND	2.6
NGO-a700	8.1	ND	ND	4.2
NGO-a900	6.7	ND	ND	7.7

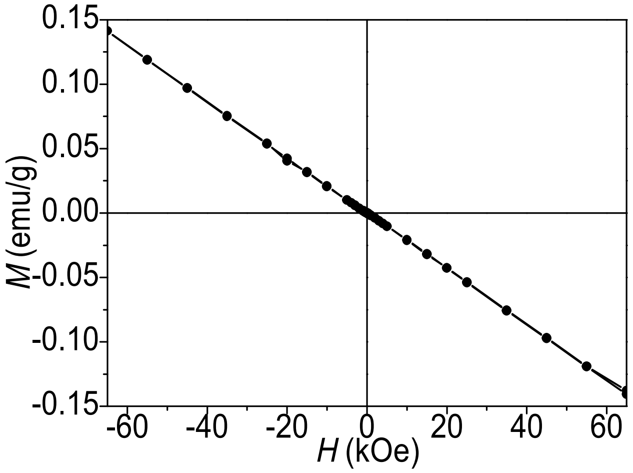

Supplementary Figure S1 | Mass magnetization (*M* – *H*) curve of NGO measured by SQUID at 300 K. It showed that no ferromagnetism can be observed, and only a purely diamagnetism can be found.

Supplementary Table S2 | The O content of GO and annealed GO samples.

Samples (at. %)	GO	RGO-300	RGO-500	RGO-700	RGO-900
O content	48.37	13.35	8.87	7.16	5.70

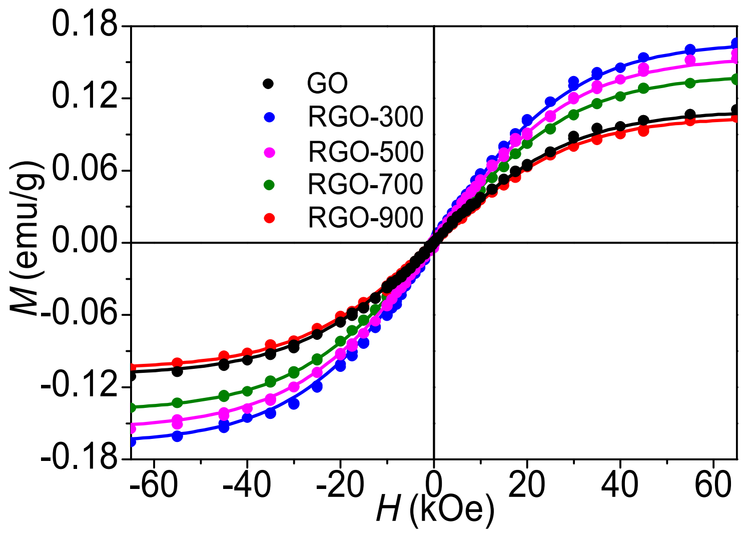

Supplementary Figure S2 | *M* – *H* curves of annealed GO samples obtained at different annealing temperatures measured by SQUID at 2 K. It is found that also no significant ferromagnetic signal is observed in all the annealed GO sampls even at 2 K. Symbols are the measurements of all the annealed GO samples and solid curves are fits to the Brillouin function of Equation (1). As shown by the fitting curves, the Brillouin function provides good fits for g= 2 and $J = S$=1/2, indicating the intrinsic spin-half paramagnetism. Also by fitting the corresponding *M-H* curves, M_s can be obtained which is 0.167 emu/g for GO–a300, 0.15 emu/g for GO–a500, 0.136 emu/g for GO–a700, and 0.102 emu/g for GO–a900, respectively.

Supplementary Table S3 | The contents of three N types and total N of the as-prepared NGO and annealed NGO samples.

Samples (at. %)	N content	N-6 content	N-5 content	N-Q content
NGO	8.80	3.45	4.00	1.35
NGO-a700	7.40	3.04	2.38	2.00
NGO-a900	6.85	2.28	2.23	2.33

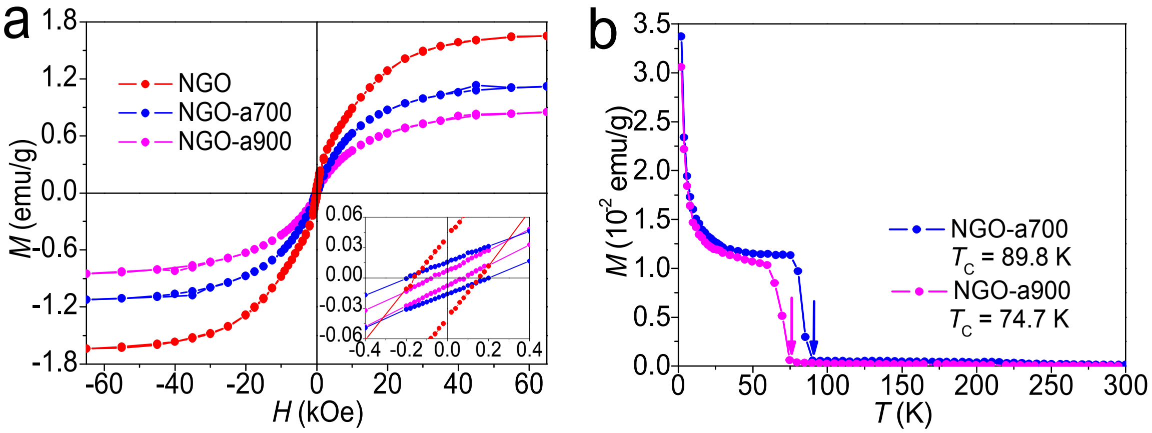

Supplementary Figure S3 | Magnetic properties of NGO and annealed NGO samples measured by SQUID. (**a**) Typical $M-H$ curves measured at 2 K. It is found both of the two annealed samples still are ferromagnetism. (**b**) $M-T$ curves of NGO-a700 and NGO-a900 measured from 2 to 300 K under the applied field H = 500 Oe. It is found that the T_C is ca. 89.8 K for NGO-a700 and 74.7 K for NGO-a900, respectively, lower than 100.2 K of the as-prepared NGO.

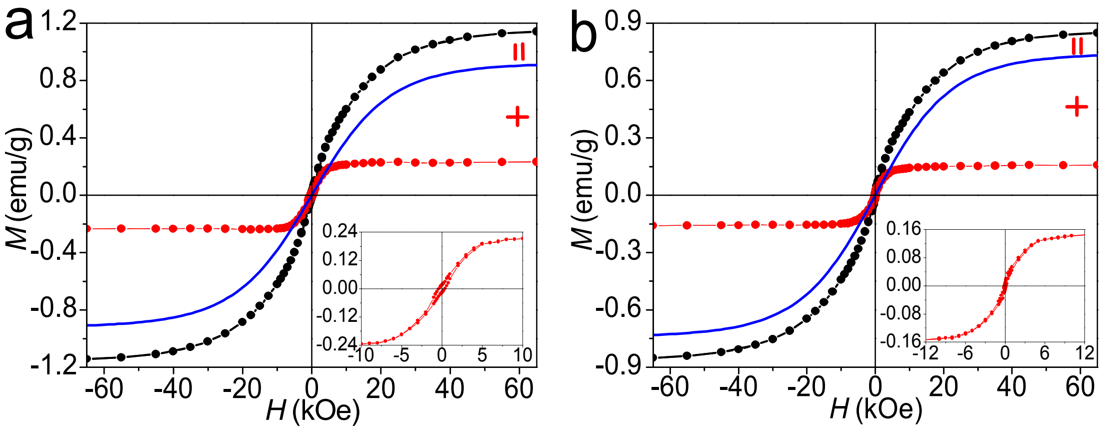

Supplementary Figure S4 | Fitting 2 K *M-H* curves of NGO-a700 (a) and NGO-a900 (b). The black curve is the measured curves and the blue lines are the fitting curves for paramagnetism by g=2 and *J* =1. The red curves are ferromagnetic mass magnetization by subtracting the paramagnetism from the measured curves. Insets

are the parts of ferromagnetic mass magnetization. The mass magnetization of NGO-a700 and NGO-a900 measured also can be expressed as $M_{\text{total}} = M_{\text{para}} + M_{\text{ferro}}$. The saturated M_{para} of NGO-a700 and NGO-a900 are fitted as 0.92 and 0.84 emu/g, and J is fitted to be 1 by Brillouin function. After subtracting the paramagnetic signals, one can obtain the remaining M_{ferro} of *ca.* 0.23 and 0.15 emu/g at 2 K of NGO-a700 and NGO-a900. Therefore, we can obtain the M_{s} of NGO-a700 and NGO-a900 at 2 K are 1.15 and 0.99 emu/g, respectively. Clearly, both the M_{ferro} and M_{s} of the two annealed samples are lower than that of the as-prepared NGO.
